# Urban prevalence and molecular confirmation of *Trypanosoma lewisi* in wild rats from Surabaya, Indonesia

**DOI:** 10.1016/j.ijppaw.2026.101264

**Published:** 2026-07-20

**Authors:** Ryanka Edila, Frenky Laksana Putra, April Hari Wardhana, Ainaya Luthfi Anindya, Seli Nurmayani, Muchammad Yunus, Seongjun Choe, Makoto Matsubayashi, Lucia Tri Suwanti

**Affiliations:** a Faculty of Veterinary Medicine, Universitas Airlangga, Jl. Mulyorejo, Kampus C, Surabaya, 60115, Indonesia; b Magister Student of Disease Science and Veterinary Public Health, Faculty of Veterinary Medicine, Universitas Airlangga, Jl. Mulyorejo, Kampus C Unair, Surabaya, East Java, 60115, Indonesia; c Research Center for Veterinary Science, Organization for Health, National Research and Innovation Agency (BRIN), Cibinong, Indonesia; d Division of Veterinary Parasitology, Faculty of Veterinary Medicine, Universitas Airlangga, Jl. Mulyorejo, Kampus C, Surabaya, 60115, Indonesia; e Department of Parasitology, School of Medicine, Chungbuk National University, Cheongju, Republic of Korea; f Laboratory of Veterinary Immunology, Faculty of Veterinary Medical Science, Osaka Metropolitan University, Osaka, Japan; g Institute of Tropical Diseases, Universitas Airlangga, Jl. Mulyorejo, Kampus C, Surabaya, 60115, Indonesia; h One Health Research Group, Faculty of Veterinary Medicine, Universitas Airlangga, Jl. Mulyorejo, Kampus C, Surabaya, 60115, Indonesia

**Keywords:** *Trypanosoma lewisi*, Wild rats, Infectious diseases, ITS1, Zoonosis, Indonesia

## Abstract

*Trypanosoma lewisi* is a blood parasite of wild rodents increasingly recognised as an emerging zoonotic pathogen associated with atypical human trypanosomiasis. However, epidemiological and molecular data from major Indonesian metropolitan areas remain limited. This study investigated the prevalence and molecular identity of *T. lewisi* in urban wild rats from Surabaya, Indonesia's second-largest city. A total of 100 wild rats, comprising *Rattus norvegicus* (n = 54) and *Rattus tanezumi* (n = 46), were captured across five geographic zones between February and August 2025. Infection was assessed by wet-mount examination and Dip Quick-stained blood smears, followed by PCR amplification of the ITS1 region of ribosomal DNA, Sanger sequencing, and Bayesian phylogenetic analysis. Overall microscopy-detectable *T. lewisi* clade prevalence was 13.0% (13/100; 95% CI: 7.1–21.2%), with infected rats detected in four of five sampling zones. All successfully sequenced samples were placed within the well-supported *T. lewisi* clade (PP = 1.0) in Bayesian phylogenetic analysis. These findings provide the first molecular evidence of *T. lewisi* circulating in urban wild rats in Surabaya and demonstrate that synanthropic rodents may serve as important reservoirs of this zoonotic parasite in densely populated Indonesian cities. The study highlights the need to incorporate rodent-borne trypanosomes into One Health surveillance frameworks in urban environments.

## Highlights

• First molecular survey of *Trypanosoma lewisi* in urban rats from Surabaya.
• *Trypanosoma lewisi* was detected in 13% of sampled wild rats.
• Both *Rattus norvegicus* and *Rattus tanezumi* carried *T. lewisi*.
• ITS1 sequencing confirmed all isolates within the *T. lewisi* clade.
• Findings support One Health surveillance of urban rodent trypanosomes.

## Graphical abstract

**Figure:**
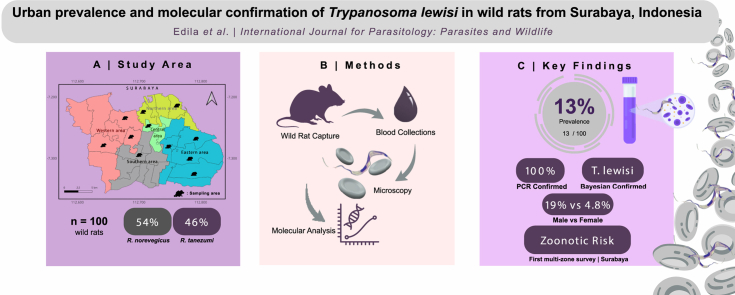

## 1 Introduction

*Trypanosoma lewisi* (Stercoraria: Trypanosomatidae) is a cosmopolitan flagellated blood parasite maintained in synanthropic rodents of the genus *Rattus*, principally *R. norvegicus*, *R. tanezumi*, *R. rattus*, and *R. exulans*, which serve as its principal reservoir hosts in human settlements worldwide (Hoare, 1972; Ortiz et al., 2018; Tanthanathipchai et al., 2023). Transmission to both rodent and incidental human hosts occurs through infected rat fleas, including *Xenopsylla cheopis* and *Nosopsyllus fasciatus* (syn. *Ceratophyllus fasciatus*), which excrete infective trypomastigotes in their faeces and contaminate skin wounds of cohabiting hosts (Nguyen et al., 2022).

Despite its historical classification as host-restricted and non-pathogenic, *T. lewisi* has increasingly been recognised as a rare zoonotic parasite capable of causing atypical human trypanosomiasis (a-HT), accounting for approximately 47% of the 21 documented a-HT cases compiled globally by Kumar et al. (2022), predominantly affecting infants and immunocompromised individuals, with clinical manifestations including fever, anaemia, and lethargy, and rare fatal outcomes (Sarataphan et al., 2007; Jain et al., 2023). Lun et al. (2015) reported that some *T. lewisi* isolates exhibit resistance to lysis by normal human serum, suggesting that reduced susceptibility to ApoL1-mediated lysis may partially explain the rare occurrence of human infections, though this property has not been demonstrated across all isolates and the underlying mechanism remains incompletely characterised.

Infection of wild rodents with *T. lewisi* and closely related members of the *T. lewisi* clade has been documented worldwide (e.g., Dobigny et al., 2011; Tang et al., 2012; Salzer et al., 2016; Archer et al., 2018; Ortiz et al., 2018; Garcia et al., 2019), with Southeast Asia identified as a region of particular epidemiological concern (Kumar et al., 2022). The ITS1 region of ribosomal DNA has been widely adopted as the primary screening marker for this clade, enabling consistent genus- and clade-level identification across studies despite ongoing taxonomic revision within the *Herpetosoma* subgenus (Ortiz et al., 2018; Egan et al., 2020). Regional prevalence in wild rodents varies considerably: 6.25% in Klang Valley, Malaysia (Mohd-Qawiem et al., 2022), 17% across human-settled communities in Thailand, Cambodia, and Lao PDR (Pumhom et al., 2014), 18% in Tha Sala district, Nakhon Si Thammarat, Thailand (Tanthanathipchai et al., 2023), and 62.5% in Hanoi, Vietnam (Nguyen et al., 2022). These prevalences have generally been reported from dense, low-sanitation settlements where human–flea–rodent contact is sustained (Pumhom et al., 2014, 2015). In Indonesia, surveys have been conducted across a limited number of localities, including Malang (Yesica et al., 2022), South Sulawesi (Winterhoff et al., 2020), Banjarnegara (Wijayanti et al., 2023), Banyuwangi (23.08%; Wardhana et al., 2024a), and East Jakarta and North Aceh (Wardhana et al., 2024b), yet major metropolitan centres remain without systematic surveillance data. Molecular methods have proved essential in this context, substantially expanding the recognised diversity of *Trypanosoma* species in rodent populations beyond what blood smear microscopy alone can detect (Ortiz et al., 2018; Egan et al., 2020).

Surabaya, the capital of East Java and Indonesia's second-largest city with a population exceeding three million (BPS, 2023), is characterised by dense coastal and riverside communities with inadequate sanitation, conditions that are highly conducive to synanthropic rodent proliferation and sustained human–rodent interface (Ramdani and Prasetyo, 2011). Despite this epidemiological significance, no surveillance data on *T. lewisi* in wild rats from Surabaya are currently available. The present study therefore aimed to characterise the prevalence, host associations, and molecular identity of *T. lewisi* across five geographic zones of Surabaya, combining blood smear microscopy, quantitative morphometrics, PCR amplification of the ITS1 region, and Bayesian phylogenetic inference.

## 2 Materials and methods

### 2.1 Study area and animal sampling

**Fig. 1:**
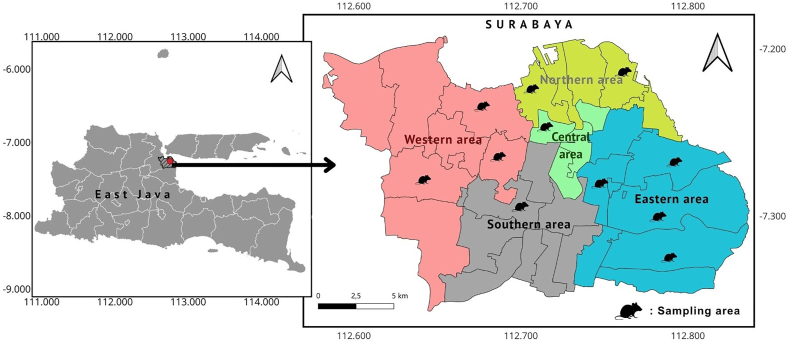
Geographic location of the study area and distribution of sampling sites. The inset map shows the position of Surabaya (red marker) within East Java Province, Indonesia. The main map delineates the five geographic sampling zones. Rat silhouettes indicate the general locations of trapping sites within each zone. Coordinate system: WGS 84.

A total of 100 wild rats were captured across five geographic zones of Surabaya: North Surabaya (Kenjeran, Bulak, and Krembangan districts), South Surabaya (Dukuh Pakis district), Central Surabaya (Bubutan and Genteng districts), East Surabaya (Sukolilo, Rungkut, Mulyorejo, and Gubeng districts), and West Surabaya (Asemrowo, Sambikerep, and Sukomanunggal districts), encompassing urban residential areas, traditional markets, and peridomestic habitats (see details in Supplementary Table 1; Fig. 1). Trapping was conducted across multiple sessions from February to August 2025. At each site, wire-mesh traps were deployed in the evening at approximately 5:00 p.m. and checked at 7:00 a.m. the following morning. Salted fish or leftover processed foods (e.g., cooked chicken) were used as bait. Trap numbers and trap-nights were not systematically recorded across sampling sessions, precluding standardisation of trapping effort and calculation of capture success. This methodological limitation should be considered when interpreting zone-level species composition and prevalence.

### 2.2 Rat identification

Captured rats were euthanised by intramuscular injection of ketamine and xylazine combination at a lethal dose, administered under institutional ethical oversight. Death was confirmed by cessation of heartbeat and respiratory movement prior to blood collection. Six external morphometric measurements were then recorded following the standard CERoPath protocol (Herbreteau et al., 2011): body weight (BW), head-body length (HB), tail length (T), hind-foot length (HF), ear length (E), and skull length (S). Sex was determined by inspection of the external and internal reproductive organs. Species identification was performed by comparing morphological and morphometric characteristics against established reference keys (Herbreteau et al., 2011; Yuliadi and Muhidin, 2016). Trapping-site variables were also recorded for each individual. Age classification was not formally performed; body weight data suggest that subadult or juvenile individuals were likely present in the sample (Supplementary Table 1), and the potential influence of age structure on species- and zone-level prevalence estimates cannot be excluded.

### 2.3 Blood collection and microscopic examination

The blood sample (1–3 mL) was collected from each rat by cardiac puncture into an EDTA blood collection tube. A drop of fresh blood was immediately placed on a glass slide, covered with a coverslip, and examined by direct microscopy at ×400 magnification. Samples showing motile trypomastigotes, characterised by the erratic displacement of surrounding erythrocytes, were recorded as positive for *Trypanosoma* spp. Simultaneously, a thin blood smear was prepared from the same blood and stained using the Dip Quick method (eosin and methylene blue; MDT IR®, Indonesia). Slides were air-dried at room temperature, fixed in methanol for 3 min, immersed successively in eosin and methylene blue solutions for 3 min each, rinsed with running water, and air-dried. Stained smears were then examined under a light microscope (Nikon Eclipse E200; Nikon Corporation, Tokyo, Japan) at ×1000 magnification under oil immersion and assessed for *Trypanosoma* spp. Key morphological structures were evaluated according to Hoare (1972), including the nucleus, kinetoplast, undulating membrane, and free flagellum. Microscopic examination was performed by one primary observer; all positive and equivocal slides were independently reviewed by two additional observers (three observers in total), with positivity recorded only upon consensus. Residual EDTA blood was stored at −20°C, and samples from individuals confirmed positive by microscopy were subsequently subjected to DNA extraction and molecular analysis.

### 2.4 Morphometric measurements

Morphometric analysis was performed on stained blood smears from all microscopically positive individuals. For each positive host, five morphologically intact trypomastigotes were measured. Only parasites with the entire body clearly visible, free of overlap with erythrocytes or other parasites, and with identifiable nucleus, kinetoplast, undulating membrane, and flagellum were included. All measurements were performed at ×1000 magnification. All measurements were obtained curvilinearly by tracing along the longitudinal body axis of each trypanosome using NIS-Elements software (Nikon Instruments Inc., Tokyo, Japan) integrated with the Nikon Eclipse E200 microscope, thereby accounting for the natural curvature of the parasite. Eleven parameters were recorded (Table 3): total body length (TL), body length exclusive of the free flagellum (BL), free flagellum length (F), nucleus length (NL), nucleus width (NW), anterior-end-to-nucleus distance (NA), nucleus-to-posterior-end distance (NP), kinetoplast-to-nucleus distance (KN), kinetoplast-to-posterior-end distance (KP), kinetoplast length (KL), and kinetoplast width (KW). All values are expressed in micrometres (μm). Descriptive statistics (mean ± SD and range) were calculated at the parasite level (n = 65) to characterise morphological variation; no inferential comparisons among host individuals were performed using morphometric data.

### 2.5 DNA extraction and PCR amplification

Genomic DNA was extracted from 300 μL of EDTA blood from each microscopically positive individual using a commercial extraction kit (Genomic DNA Mini Kit; Geneaid Biotech Ltd., New Taipei City, Taiwan) following the manufacturer's instructions. The extracted DNA was stored at −20°C until further use. Molecular detection of *Trypanosoma* spp. was carried out by conventional PCR, targeting the internal transcribed spacer 1 (ITS1) region of ribosomal DNA using the TRYP1 primer set (Desquesnes et al., 2002, 2011). This primer set yields a diagnostic amplicon of approximately 623 bp for *T. lewisi*. The primers used were TRYP1S (5′-CGT CCC TGC CAT TTG TAC ACA-3′) as the forward primer and TRYP1R (5′-GGA AGC CAA GTC ATC CAT CG-3′) as the reverse primer. Each reaction was prepared in a total volume of 25 μL, containing MyTaq™ HS Mix (Meridian Bioscience Inc., Cincinnati, OH, USA), 0.4 μM of each primer, 2 μL of template DNA, and nuclease-free water. PCR amplification was performed on a Biometra Tone thermocycler (Biometra GmbH, Göttingen, Germany) under the following conditions: initial denaturation at 95°C for 1 min; followed by 35 cycles of denaturation at 95°C for 15 s, annealing at 58°C for 15 s, and extension at 72°C for 15 s; and a final extension at 72°C for 10 min. PCR products were electrophoresed on a 1.5% agarose gel in TAE buffer at 100 V for 30 min alongside a 1000 bp DNA ladder. Gels were stained with Fluoro® Safe (1st BASE Pte. Ltd., Singapore) and visualised using a GelDoc Transluminator (Cleaver Scientific Ltd., Rugby, UK). Each PCR run included a positive control (DNA from a previously confirmed *T. lewisi*-positive blood sample) and a negative control (nuclease-free water replacing template DNA) to monitor contamination and reaction performance. For samples yielding poor-quality chromatograms, PCR amplification was repeated; however, sufficient sequence quality for consensus assembly could not be achieved in any case.

### 2.6 DNA sequencing and phylogenetic analysis

The ITS1 region was selected as the primary phylogenetic marker because it is the most widely validated locus for clade-level identification of *Trypanosoma lewisi* and related species (Desquesnes et al., 2002, 2011). Positive PCR amplicons were purified and sequenced by Sanger sequencing at PT. Genetika Science Indonesia (Tangerang, Indonesia). Raw reads were assembled, trimmed, and manually curated in Geneious Prime® 2024.0.5 (Biomatters, Auckland, New Zealand). Sequences were queried against GenBank using BLASTN (https://blast.ncbi.nlm.nih.gov; Altschul et al., 1990) for preliminary taxonomic assignment and identification of closest matching sequences in the database. Newly generated sequences were aligned with published reference sequences using MAFFT as implemented in Geneious Prime; the alignment was trimmed to the shortest sequence and ambiguously aligned positions were excluded manually. The final alignment comprised 363 nucleotide positions.

The best-fit nucleotide substitution model was selected using jModelTest v2.1.10 (Darriba et al., 2012) under the Bayesian Information Criterion (BIC), which identified the HKY model. Bayesian phylogenetic inference was conducted in MrBayes v3.2.6 (Ronquist et al., 2012) with four Markov chains run for 20,000,000 generations (sampled every 1000 generations; first 25% of trees discarded as burn-in). *Leishmania donovani* (GenBank accession no. AM901453; Alam et al., 2009) served as the outgroup. The consensus tree was visualised in FigTree v1.4.4 (Rambaut, 2012). Uncorrected pairwise distances (p-distance; Nei and Kumar, 2000) were calculated in MEGA v11 (Tamura et al., 2021).

### 2.7 Statistical analysis

Infection prevalence was expressed as a percentage with exact 95% confidence intervals (CIs) calculated using the Clopper–Pearson method (Clopper and Pearson, 1934). Differences in prevalence across host species, sex, and sampling zone were assessed by the chi-square test or, when expected cell counts were below five, by Fisher's exact test (Fisher, 1935). A p-value of less than 0.05 was considered statistically significant. Morphometric data are presented as mean ± SD and range (minimum–maximum). All statistical analyses were performed in SPSS v26.0 (IBM Corp., Armonk, NY, USA).

## 3 Results

### 3.1 Rodent capture and species composition

A total of 100 wild rats were captured across five geographic zones of Surabaya between February and August 2025. Morphological and morphometric identification resolved two species: *Rattus norvegicus* (n = 54; 54%) and *Rattus tanezumi* (n = 46; 46%). *R. norvegicus* was the predominant species in North (25/30) and West Surabaya (6/10), whereas *R. tanezumi* was more frequently recovered from Central (13/23) and East Surabaya (17/28). Capture sites spanned residential areas and traditional markets across all five zones. Individual rat data and trapping-site variables are provided in Supplementary Table 1.

### 3.2 Microscopic detection

**Fig. 2:**
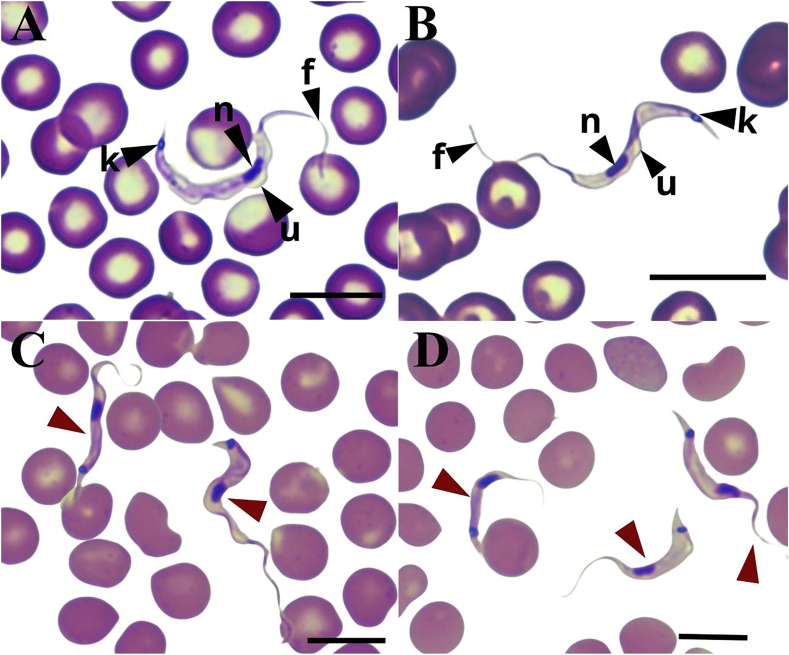
Blood-stage trypomastigotes of *Trypanosoma lewisi* in Dip Quick-stained thin blood smears from naturally infected *Rattus* spp. captured in Surabaya, Indonesia. (A, B) Morphological details showing nucleus (n), kinetoplast (k), undulating membrane (u), and flagellum (f). (C, D) Representative fields showing multiple trypomastigotes (arrowheads) among host erythrocytes. Scale bars = 10 μm.

Wet-mount direct examination and Dip Quick-stained thin blood smear evaluation each identified 13 of 100 individuals as positive for blood-stage trypomastigotes, establishing complete concordance between the two techniques. In stained preparations, parasites were morphologically consistent with *Trypanosoma lewisi* Hoare (1972): a pointed posterior end, subterminal oval kinetoplast, anteriorly positioned nucleus, well-developed undulating membrane, and a free anterior flagellum (Fig. 2). All 87 remaining samples were negative by both techniques.

### 3.3 Infection prevalence and associated host factors

The overall prevalence of microscopy-detectable *T. lewisi* clade infection was 13.0% (13/100; 95% CI: 7.1–21.2%). Infection was detected in four of the five sampling zones (Table 1), with the highest rate recorded in South Surabaya (22.2%, 2/9; 95% CI: 2.8–60.0%), followed by North Surabaya (20.0%, 6/30; 95% CI: 7.7–38.6%), Central Surabaya (17.4%, 4/23; 95% CI: 5.0–38.8%), and West Surabaya (10.0%, 1/10; 95% CI: 0.3–44.5%). No positive individuals were detected in East Surabaya (0/28).

**Table 1:** Prevalence of *Trypanosoma lewisi* in wild rats by sampling area and species in Surabaya, Indonesia.

Sampling Area	Species	Examined (n)	Positive (n)	Prevalence (%)	95% CI
North Surabaya	*R. norvegicus*	25	5	20	6.8–40.7
	*R. tanezumi*	5	1	20	0.5–71.6
	**Subtotal**	**30**	**6**	**20**	**7.7**–**38.6**
South Surabaya	*R. norvegicus*	2	1	50	1.3–98.7
	*R. tanezumi*	7	1	14.3	0.4–57.9
	**Subtotal**	**9**	**2**	**22.2**	**2.8**–**60.0**
Central Surabaya	*R. norvegicus*	10	1	10	0.3–44.5
	*R. tanezumi*	13	3	23.1	5.0–53.8
	**Subtotal**	**23**	**4**	**17.4**	**5.0**–**38.8**
East Surabaya	*R. norvegicus*	11	0	0	0.0–28.5
	*R. tanezumi*	17	0	0	0.0–19.5
	**Subtotal**	**28**	**0**	**0**	**0.0**–**12.3**
West Surabaya	*R. norvegicus*	6	1	16.7	0.4–64.1
	*R. tanezumi*	4	0	0	0.0–60.2
	**Subtotal**	**10**	**1**	**10**	**0.3**–**44.5**
**Total**	**–**	**100**	**13**	**13**	**7.1**–**21.2**

95% CI = 95% confidence interval (Clopper–Pearson exact method).

Prevalence in *R. norvegicus* (14.8%, 8/54; 95% CI: 6.6–27.1%) was comparable to that in *R. tanezumi* (10.9%, 5/46; 95% CI: 3.6–23.6%), with no significant difference between species (p = 0.767). Male rats were infected at a higher rate (19.0%, 11/58; 95% CI: 9.9–31.4%) than females (4.8%, 2/42; 95% CI: 0.6–16.2%), a difference that did not reach statistical significance (p = 0.067; Table 2). Fisher's exact test was applied for all comparisons, as expected cell counts were below five in several categories.

**Table 2:** Association between *Trypanosoma lewisi* infection and host variables (species and sex) in wild rats from Surabaya, Indonesia.

Variable	Category Area	Examined (n)	Positive (n)	Prevalence (%)	95% CI	p-value
Species	*R. norvegicus*	54	8	14.8	6.6—27.1	0.767
	*R. tanezumi*	46	5	10.9	3.6—23.6	
Sex	Male	58	11	19	9.9—31.4	0.067
	Female	42	2	4.8	0.6—16.2	
**Total**	**—**	**100**	**13**	**13**	**7.1—21.2**	

95% CI = 95% confidence interval (Clopper–Pearson exact method).

**Table 3:** Morphometric measurements of *Trypanosoma lewisi* bloodstream trypomastigotes detected in wild rats from Surabaya, Indonesia (n = 65).

Parameter	Mean ± SD	Range
Total body length (TL; μm)	31.84 ± 2.75	18.25–35.79
Body length without flagellum (BL; μm)	23.74 ± 2.96	12.56–27.59
Free flagellum length (F; μm)	8.06 ± 1.48	5.52–12.51
Nucleus length (NL; μm)	2.65 ± 0.41	2.00–3.98
Nucleus width (NW; μm)	0.82 ± 0.16	0.58–1.58
Anterior end to nucleus distance (NA; μm)	9.66 ± 2.24	3.15–15.08
Nucleus to posterior end distance (NP; μm)	14.09 ± 2.00	9.35–16.83
Kinetoplast to nucleus distance (KN; μm)	10.09 ± 1.50	5.72–12.93
Kinetoplast to posterior end distance (KP; μm)	4.03 ± 1.00	1.78–6.63
Kinetoplast length (KL; μm)	0.96 ± 0.13	0.65–1.34
Kinetoplast width (KW; μm)	0.89 ± 0.16	0.58–1.29

### 3.4 Molecular confirmation and sequence identification

PCR with the TRYP1S/TRYP1R primer pair, anchored in the flanking 18S (TRYP1S) and 5.8S (TRYP1R) rRNA genes and targeting the complete ITS1 region of the ribosomal DNA array, was applied to all 13 microscopically positive individuals, each yielding a single ∼623 bp amplicon (Desquesnes et al., 2002). All 13 amplicons were submitted for Sanger sequencing; seven met the quality and length thresholds for phylogenetic analysis, whereas the remaining six produced low-quality chromatograms that precluded sequence assembly and were therefore excluded. Preliminary BLASTN searches of these partial reads indicated highest similarity to *T. lewisi* reference sequences. Among the seven phylogeny-grade sequences, BLASTN comparison identified all as *T. lewisi* (E-value = 0.0), with highest nucleotide identity to EU861192.1 (*T. lewisi* 18S rRNA gene, partial; ITS1, complete; 620 bp) ranging from 98.68% to 99.81% (query coverage: 100%). The seven sequences have been deposited in GenBank under accession numbers PZ382750-PZ382756.

### 3.5 Phylogenetic analysis

**Fig. 3:**
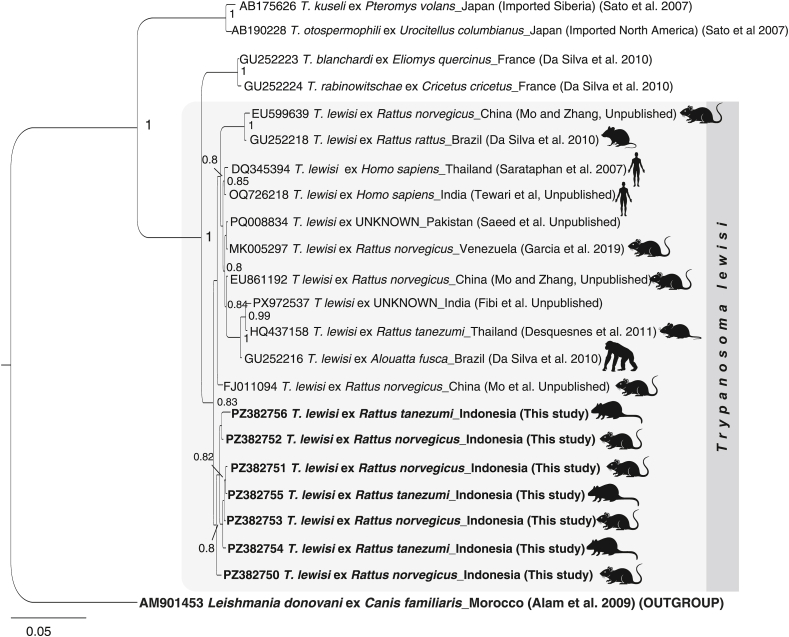
Bayesian inference (BI) phylogenetic tree based on the ITS1 region of the ribosomal DNA array (18S–ITS1–5.8S) from *Trypanosoma lewisi* and related species. Sequences obtained from *Rattus norvegicus* and *Rattus tanezumi* in the present study are shown in bold. The tree was inferred using the HKY substitution model with 20,000,000 MCMC generations (sampled every 1000 generations; first 25% discarded as burn-in). Posterior probability values equal to or greater than 0.80 are shown at the nodes. *Leishmania donovani* (GenBank: AM901453) was used as the outgroup. The scale bar represents the number of nucleotide substitutions per site.

Bayesian phylogenetic analysis of the seven ITS1 sequences together with GenBank reference sequences placed all study sequences within the strongly supported *T. lewisi* clade (PP = 1.00). The final trimmed alignment comprised 363 nucleotide positions. Within this clade, the study sequences formed a moderately supported monophyletic subgroup (PP = 0.83) and were interspersed with reference sequences from diverse hosts, including *Rattus norvegicus*, *Rattus tanezumi*, and *Homo sapiens*, and multiple geographic regions (Indonesia, India, China, Thailand, Venezuela, and Brazil), with no apparent clustering by host or geographic origin. The *T. lewisi* clade was clearly separated from related species, including *T. blanchardi*, *T. rabinowitschae*, *T. kuseli*, and *T. otospermophili*, as well as the outgroup *Leishmania donovani* (AM901453) (Fig. 3).

Uncorrected pairwise p-distances among the seven study sequences ranged from 0 to 0.027; three sequences (PZ382751, PZ382755, and PZ382754) were identical (p-distance = 0). Distances between the study sequences and other *T. lewisi* reference entries in the dataset ranged from 0.011 to 0.069. In contrast, p-distances separating the *T. lewisi* clade from the outgroup *Leishmania donovani* ranged from 0.47 to 0.49. The complete pairwise distance matrix is provided in Supplementary Table 2.

### 3.6 Morphometric measurements

Morphometric data are presented as supportive evidence of consistency with previously described *T. lewisi* clade trypomastigotes and are not independently diagnostic of species identity. Morphometric measurements were obtained from all 13 confirmed individuals (five measurements per individual; n = 65), covering 11 structural parameters (Table 3). Mean total body length (TL) was 31.84 ± 2.75 μm (range: 18.25–35.79 μm). The free flagellum (F: 8.06 ± 1.48 μm) constituted approximately 25% of TL. The nucleus was anteriorly positioned at a mean distance of 9.66 ± 2.24 μm from the anterior tip (NA), with mean dimensions of 2.65 ± 0.41 μm (NL) and 0.82 ± 0.16 μm (NW). The kinetoplast was small and oval, located at 4.03 ± 1.00 μm from the posterior end (KP) and 10.09 ± 1.50 μm from the nucleus (KN), measuring 0.96 ± 0.13 μm (KL) and 0.89 ± 0.16 μm (KW). All parameters were consistent with morphometric bounds established for *T. lewisi* by Hoare (1972).

## 4 Discussion

The present study constitutes the first multi-zone epidemiological and molecular characterisation of *Trypanosoma lewisi* in Surabaya, an approach that remains uncommon in Indonesian rodent parasite surveys and is particularly warranted for a metropolitan centre of this demographic scale. The overall prevalence of microscopy-detectable infection was 13.0% (95% CI: 7.1–21.2%), consistent with the range documented across urban rodent populations in Southeast Asia: 18% in Nakhon Si Thammarat (Tanthanathipchai et al., 2023) and 17% across human-settled communities in Thailand, Cambodia, and Lao PDR (Pumhom et al., 2014). Direct comparison of point estimates, however, requires strict attention to diagnostic protocol, a variable that accounts for a disproportionate share of inter-site variance. Studies applying universal PCR yield systematically higher rates: 23.08% and 24.26% by smear and PCR respectively in Banyuwangi (Wardhana et al., 2024a), 62.5% in Hanoi, Vietnam (Nguyen et al., 2022), and 46.15–66.67% across residential and livestock environments in East Jakarta and North Aceh (Wardhana et al., 2024b). In the present study, PCR was applied exclusively to the 13 microscopically confirmed individuals; Yudhana et al. (2025) demonstrated that this sequential approach underestimates true molecular prevalence relative to universal TRYP1 screening, particularly for low-parasitaemia infections below the microscopy threshold (Desquesnes et al., 2011). The 13.0% figure reported here is therefore a conservative lower bound on the actual infection rate in Surabaya's urban rat population.

Zone-level prevalence ranged from 0% in East Surabaya (0/28; 95% CI: 0.0–12.3%) to 22.2% in South Surabaya (2/9; 95% CI: 2.8–60.0%), though the non-probability trapping design and pronounced imbalance in zone sample sizes (9 to 30 individuals per zone) preclude robust causal inference about spatial determinants of infection. The zero prevalence in East Surabaya, despite the largest zone sample, should be regarded as a signal requiring structured follow-up rather than confirmed absence; the upper confidence bound (12.3%) retains epidemiological relevance. Pronounced within-city microhabitat variation in *T. lewisi* prevalence has been documented in comparable settings; Wardhana et al. (2024b) recorded rates of 66.67% in indoor-trapped versus 28.57% in outdoor-trapped rats within a single North Aceh locality. These external findings suggest that habitat-stratified sampling would more effectively resolve the spatial heterogeneity of infection risk than zone-level aggregation, and represent a methodological priority for future surveys in Surabaya. Additionally, the absence of systematic trapping effort records precludes calculation of trap-night-standardised capture rates, limiting inference about zone-level differences in rodent density.

Both *Rattus norvegicus* and *Rattus tanezumi* were infected at statistically indistinguishable rates (14.8% vs. 10.9%; p = 0.767), consistent with the broad intra-generic host range of *T. lewisi* within the tribe Rattini established by Hoare (1972). This inter-specific equivalence in Surabaya contrasts with findings from Banyuwangi, where *R. tanezumi* was more frequently infected than *R. norvegicus* (Wardhana et al., 2024a), and with the Jakarta and North Aceh data in which *R. tanezumi* accounted for 62.07% of captures and appeared to dominate the peridomestic reservoir community (Wardhana et al., 2024b). Such inter-site discordance is most parsimoniously attributed to local variation in species abundance, spatial overlap with *Xenopsylla cheopis* populations, and trapping microhabitat rather than intrinsic differences in host susceptibility (Pumhom et al., 2015; Wardhana et al., 2024a). In the present study, the near-equal representation of both species across residential areas and traditional markets likely resulted in comparable flea exposure, precluding detection of a species-level prevalence difference even if one exists in the broader population. That both species are competent hosts and active reservoir candidates in Surabaya carries direct surveillance implications: neither species warrants exclusion from rodent monitoring programs targeting the rodent-flea-human transmission interface. Species identification of the captured rodents relied exclusively on external morphometric measurements and morphological characteristics following the CERoPath protocol, without molecular confirmation. Given the occurrence of morphologically cryptic Rattus species in Southeast Asia and the absence of molecular host species confirmation in the present study, species assignments and species-level prevalence comparisons should be interpreted with considerable caution.

Male rats showed a higher infection rate than females (19.0%, 11/58 vs. 4.8%, 2/42; p = 0.067), a non-significant trend whose direction is nonetheless consistent with sex-disaggregated patterns reported in other urban rat populations, where males have been found to carry higher ectoparasite burdens and engage in broader-ranging behaviour associated with elevated flea-vector exposure (Linardi et al., 1985; Linardi and Botelho, 2002; Wardhana et al., 2024a). The failure to reach statistical significance is most plausibly attributable to limited power: with only two infected females among 42 sampled, the dataset cannot reliably distinguish a true sex-associated risk difference from sampling variance. A prospective sex-stratified design with adequate representation of positive cases in both groups would be needed to resolve this association.

PCR confirmed the identity of all 13 microscopy-positive samples; however, because microscopy-negative animals were not tested, the sensitivity of the sequential microscopy-based screening approach could not be assessed. The necessity of sequence-level identification is underscored by the morphological similarity between *T. lewisi* trypomastigotes and those of other *Trypanosoma* species reported in rodents, which renders morphology alone an insufficient basis for species assignment in field surveys (Desquesnes et al., 2011, 2022; Ortiz et al., 2018). All 13 sequences returned highest identity to *T. lewisi* (E-value = 0.0); among the seven phylogeny-grade sequences, nucleotide identity to EU861192.1 (*T. lewisi* 18S rRNA gene, partial; ITS1, complete; 620 bp) ranged from 98.68% to 99.81% (query coverage: 100%), consistent with the high inter-isolate similarity characteristic of this locus (Desquesnes et al., 2011; Yesica et al., 2024).

Bayesian phylogenetic inference positioned all seven high-quality sequences in a monophyletic subgroup (PP = 0.83) within a strongly supported *T. lewisi* clade (PP = 1.0), with study sequences distributed throughout the clade alongside reference sequences from *R. norvegicus*, *R. tanezumi*, and *Homo sapiens* from Indonesia, India, China, Thailand, Venezuela, and Brazil, without clustering by host species or geographic origin. This phylogeographic homogeneity reflects the inherently limited intraspecific resolving power of the ITS1 locus (Desquesnes et al., 2011) and the cosmopolitan dispersal of *T. lewisi* via global *Rattus* translocation (Hoare, 1972; Puckett et al., 2020). Three sequences from North Surabaya (PZ382753) and Central Surabaya (PZ382754, PZ382755) were identical (p-distance = 0), a result compatible with recent inter-zone transmission or shared recent ancestry within the urban rat metapopulation; resolution between these interpretations requires the higher intraspecific resolution afforded by the nine microsatellite loci developed specifically for *T. lewisi* (Segard et al., 2022). It is acknowledged that ITS1 alone has inherent limitations for species delimitation within the *T. lewisi* complex, where sibling taxa share near-identical sequences; formal resolution would require a multi-locus approach incorporating additional markers such as hsp70 or 18S rRNA.

Morphometric measurements from all 13 confirmed individuals conform to the canonical description of *T. lewisi* (Hoare, 1972). The key positional parameters, kinetoplast-to-posterior-end distance (KP: 4.03 ± 1.00 μm) and kinetoplast-to-nucleus distance (KN: 10.09 ± 1.50 μm), closely match values from *T. lewisi* detected in a human case from Thailand (KP: 4.2 ± 2.8 μm; KN: 9.5 ± 2.8 μm; Sarataphan et al., 2007), confirming cross-regional morphological consistency of the species in peridomestic urban hosts. The wide range in total body length (18.25–35.79 μm) reflects intraspecific pleomorphism rather than measurement artefact. The minimum value originated from host T72, whose five trypomastigotes were consistently shorter (mean TL = 24.78 μm vs. 30.13–33.79 μm in the remaining hosts) and showed proportionally reduced dimensions, indicating predominance of the stumpy form, consistent with previous observations (Tanthanathipchai et al., 2023). These measurements provide baseline morphometric data for *T. lewisi* clade trypomastigotes from urban rats in Surabaya and may serve as a reference for future studies in Indonesia, although morphology alone cannot confirm species identity within the clade.

These findings should be contextualised within the growing evidence base for *T. lewisi* as an emerging zoonotic pathogen. Kumar et al. (2022) compiled 21 documented atypical human trypanosomosis cases globally, of which 10 (47%) were attributable to *T. lewisi*, all from Asia, with a disproportionate share among neonates and immunocompromised individuals (Sarataphan et al., 2007; Jain et al., 2023). That some isolates of organisms within the *T. lewisi* clade may partially resist lytic factors in normal human serum (Lun et al., 2015) indicates that susceptibility may not be strictly confined to immunocompromised hosts, though the conditions required for productive human infection remain incompletely defined and warrant prospective investigation.

Accumulating evidence indicates that the reservoir community for *T. lewisi* may extend beyond commensal *Rattus* spp. Villalba-Aleman et al. (2024) detected *T. lewisi*-like trypanosomes in synanthropic bats roosting within households in Brazil and Venezuela, and Babyesiza et al. (2024) documented spillover into non-commensal native small mammals at urban-wildlife interfaces in Uganda. These findings suggest that multi-taxon surveillance approaches would provide a broader characterisation of *T. lewisi* transmission ecology than rodent-focused surveys alone can offer.

The detection of *T. lewisi* across four of five sampling zones in Surabaya, a metropolitan centre of over three million inhabitants where no routine surveillance for rodent-borne trypanosomes currently exists, highlights a gap in the regional evidence base that warrants structured follow-up. These findings support incorporating *T. lewisi* into One Health urban rodent monitoring frameworks and raising clinical awareness among practitioners managing febrile illness in high-risk populations (Kumar et al., 2022; Wardhana et al., 2024a). Future studies employing universal PCR-based screening alongside concurrent vector-arthropod surveillance would substantially strengthen epidemiological inference beyond what the present sequential microscopy-confirmation approach can provide.

## Ethical approval

All animal procedures were approved by the Ethics Committee of the Faculty of Veterinary Medicine, Airlangga University, Surabaya, Indonesia (approval number: 2.KEH.15.02.2025) and conducted in accordance with the applicable institutional guidelines for the care and use of animals.

## Data availability

The representative sequences obtained in this study were deposited in GenBank under the following accession numbers: PZ382750-PZ382756.

## CRediT authorship contribution statement

**Ryanka Edila:** Conceptualization, Data curation, Formal analysis, Software, Validation, Visualization, Writing – original draft. **Frenky Laksana Putra:** Data curation, Investigation. **April Hari Wardhana:** Conceptualization, Methodology, Resources, Supervision, Validation. **Ainaya Luthfi Anindya:** Data curation, Investigation. **Seli Nurmayani:** Data curation, Investigation. **Muchammad Yunus:** Project administration, Supervision. **Seongjun Choe:** Validation, Writing – review & editing. **Makoto Matsubayashi:** Validation, Writing – review & editing. **Lucia Tri Suwanti:** Conceptualization, Funding acquisition, Methodology, Project administration, Supervision, Validation, Writing – review & editing.

## Declaration of competing interests

The authors declare that they have no known competing financial interests or personal relationships that could have appeared to influence the work reported in this paper.
